# Organic Matter in Air

**Published:** 1880-11

**Authors:** 


ORGANIC MATTER IN THE AIR.

In the *National Board of Health Bulletin* for September 11th, 1880, is an interesting report on this subject by Ira Remsen.

The author of the report describes fully his methods of investigation, but points out the many difficulties to be encountered, and acknowledges the inconclusive nature of his results. The whole report is valuable as a step in the right direction, but is too lengthy for copying in full. The following, however, are his conclusions and suggestions:

"In looking back over the field traversed I cannot avoid a feeling of dissatisfaction. A great deal of time has been spent in this investigation. Constant attention to minute details has been necessary, and at times disappointment has followed disappointment. Here and there, however, light has been thrown upon the subject; and whatever its future development may be, I think the facts established here will always stand. Let us, then, pass quickly in review the main results.

"1. The nitrogenous matter of the air may be thoroughly collected by means of the pumice-stone absorber described in this report.

"2. The total amounts of ammonia found in experiments performed at the same time with the same specimens of air, agree fairly well with one another; so much so as to warrant the use of the method for the examination of the air.

"3. When free and albuminoid ammonia are determined, the results obtained do not always agree very closely, but still the agreement is sufficient to enable the experimenter to detect such variations as are likely to occur between pure and impure air.

"4. Air contaminated by being drawn through water containing decaying meat, does not yield more than the usual quantity of albuminoid ammonia.

"5. Air contaminated by being drawn over comparatively dry decaying organic matter, yields more than the usual quantity of albuminoid ammonia.

"6. Air contaminated by respiration, yields more than the usual quantity of albuminoid ammonia.

"7. It is necessary in judging of the purity of air to take all the facts known in regard to it into consideration. The simple determination of any one constituent can never be a sufficient basis for the formation of a competent judgment.

"8. It would be useless to have examinations of air made by any but the most careful workers. It would be time thrown away to have such analyses made by the average practical chemist.

"Finally, as to suggestions for the future. A number of questions have been left unanswered in this investigation. A very important one is this: Is the air which has been deprived of its nitrogenous matter also deprived of injurious constituents? If this is ever to be answered, it is evident that it cannot be by chemical methods. The effect of the air on fermentable liquids must be studied, and also on animals. Can an animal continue to breathe normally in such air?

"'A second question which ought to be answered is this: Does the amount of organic matter in the air vary with different conditions of the air, as for instance, with the hygrometric state? This can only be answered by a long-continued, systematic series of examinations of the air, such as are at present being made at Glasgow, Montsouris, and at some places in Germany. It may be said, however, that, up to the present, no connection has been observed between the amount of organic matter and other conditions of the air.

"'Again, it would be interesting to determine how much nitrogenous organic matter from ordinary contaminating sources can be held in suspension in the air.

"Many other questions suggest themselves, but those given above indicate that an almost unlimited field opens before us, and show clearly that the only way in which results of great value can be reached is by united effort. A single individual can do little more than point out the way in which results may be reached, and clear the road for advances. It will only be when health departments in a great many places are impressed with the importance of such subtle examinations as those of the air, and provide the means for making them through long periods, that our knowledge will be materially increased on the subject treated in this report. When records, made year after year by competent men using the best methods, are in our possession, then probably conclusions of great importance for sanitary science will be drawn."'

---

IN consequence of a singular error in proof-reading, the name of the author of the interesting lecture on the treatment of urethral stricture by the "perineal section," published in our last issue, was announced as L. Ranney. We take this opportunity of stating that the contributor who kindly favored us with the production referred to was Professor Ambrose L. Ranney, Adjunct Professor of Anatomy in the University of the City of New York, Medical Department.

